# History Is the Key to Diagnosis: A Case of Nitrofurantoin-Induced Interstitial Lung Disease

**DOI:** 10.7759/cureus.56097

**Published:** 2024-03-13

**Authors:** Kristina Akopyan, Raaed Zafar, Ibrahim Faruqi

**Affiliations:** 1 Internal Medicine, University of Florida, Gainesville, USA; 2 Medicine, University of Texas at Dallas, Dallas, USA; 3 Pulmonary and Critical Care Medicine, University of Florida, Gainesville, USA

**Keywords:** ground-glass opacity, residual volume, total lung capacity, restrictive lung disease, nitrofurantoin, interstitial lung disease

## Abstract

We present the case of a 74-year-old woman with a past medical history (PMH) significant for anxiety, depression, and hypertension who presented to the pulmonary clinic for consultation regarding progressive shortness of breath, which started five months ago after developing COVID-19. Further history-taking revealed that she had been started on nitrofurantoin two months ago for recurrent urinary tract infections (UTIs). Her pulmonary function tests (PFTs) demonstrated a moderately restrictive disease. A CT chest was obtained, showing pleural thickening with bilateral pleural-based ground glass opacities. Nitrofurantoin was then discontinued, and she was started on a prednisone taper for suspected nitrofurantoin-induced interstitial lung disease (ILD). At a follow-up clinic visit six months later, she showed great improvement in her shortness of breath, marked improvement in forced vital capacity (FVC) on PFTs, and near resolution of pleural-based lesions and basal ground glass opacities on CT chest. This case emphasizes the importance of keeping the diagnosis of nitrofurantoin-induced ILD in mind, as well as the need to implement guidelines for the monitoring of this potential pulmonary adverse effect.

## Introduction

Nitrofurantoin is a recommended first-line antibiotic medication primarily used in the treatment and prophylaxis of uncomplicated urinary tract infections (UTIs). The drug is activated by bacterial enzymes to a reduced form, which binds to the bacterial ribosomes. This reduced form inhibits protein synthesis and consequently affects bacterial DNA, RNA, and cell wall synthesis [1]. Nitrofurantoin can potentially cause marked or irreversible pulmonary complications. The complications can be categorized into an acute hypersensitivity reaction, occurring soon after a short course of the medication, or a chronic pulmonary reaction, which develops after months to years of nitrofurantoin therapy [2,3]. The data from studies conducted to assess nitrofurantoin-induced lung injury suggests that nitrofurantoin can directly injure normal lung parenchymal cells, probably through oxidant mechanisms or indirectly by association with the recruitment of neutrophils to the lung parenchyma [4]. This case presents a patient who developed nitrofurantoin-induced ILD, which is a rare pulmonary reaction associated with long-term nitrofurantoin therapy. ILD refers to a group of diffuse parenchymal lung diseases with similar clinical, radiographic, physiologic, or pathologic manifestations associated with extensive alteration of alveolar and airway architecture [5]. Discontinuation of nitrofurantoin and the initiation of a steroid taper resulted in the subsequent improvement of shortness of breath in our patient. This case demonstrates the current need to put forth guidelines outlining the timely monitoring and screening of nitrofurantoin-induced pulmonary adverse effects.

## Case presentation

A 74-year-old woman with a past medical history (PMH) significant for anxiety, depression, and hypertension presented to the pulmonary clinic for consultation regarding progressive shortness of breath, which started five months ago after the onset of COVID-19. At the start of her infection with COVID-19, she had symptoms of fatigue, sneezing, and coughing. She described coughing up a small amount of sputum sometimes in the morning. She received a course of Paxlovid while staying at home. She received a full vaccination, including a booster shot against COVID-19. She reported having allergies in the past but denied any prior history of shortness of breath. She quit smoking tobacco 40 years ago and had a 15-pack-year history of smoking tobacco. Her dyspnea continued to progress, and she could only walk a maximum of 400 feet on a good day.

She was asked about potential respiratory exposures that may be relevant to ILD. She denied a history of exposure to hazardous materials such as asbestos, coal, fiberglass, organic dust, inorganic dust, volatile chemicals, spray paints, pigeons or other birds, a farming environment, humidifiers, and hot tubs. Regarding the possibility of occult connective tissue disease, her history is remarkable for reflux disease. Her history is negative for joint pain, joint swelling, joint redness, skin rash, photosensitivity, mouth ulcers, fingertip ulcers, muscle pains, or tightening of the skin on the hands and face. Regarding the possibility of sarcoidosis, her history is negative for renal stones, eye disease, syncope, palpitations, heart failure, or hypercalcemia. Further history-taking revealed that she had been started on nitrofurantoin two months ago for recurrent UTIs. The patient was on nitrofurantoin 100 mg daily for two months.

During the physical examination, her oxygen saturation was 90% on room air, her blood pressure was 127/72, her heart rate was 99, and her temperature was 97.6 °F. She was well-developed and in no acute distress. She had no sinus tenderness, and her oral pharynx was clear without exudate or drainage. Pupils were equal, round, and reactive to light. There was no palpable cervical or supraclavicular lymphadenopathy. She had faint crackles at the base of her lungs. S1 and S2 were normal, with no murmurs noted. The abdomen was non-distended, with no tenderness. The skin was warm and dry, with no rashes noted. There was no clubbing or cyanosis. Pulmonary function tests (PFTs) revealed moderately restrictive disease (Table 1).

**Table 1:** Pulmonary function tests at initial clinic visit LLN: lower limit of normal; ULN: upper limit of normal; Pre: prior to bronchodilator; Pre%Pred: ratio of patient's actual results compared to predicted normal values; FVC: forced vital capacity; FEV: forced expiratory volume; TLC: total lung capacity; VC: vital capacity; RV: residual volume; DLCO: diffusion capacity of the lungs for carbon monoxide

	LLN	ULN	Pre	Pre%Pred
FVC (L)	1.75	-	1.46	60.1
FEV 0.5 (L)	0.5	0.65	1.46	97.3
TLC (L)	3.45	5.42	2.91	65.6
VC (L)	1.47	2.86	1.83	84.7
RV (L)	1.41	2.56	1.08	54.1
DLCO mL/(min*mmHg)	12.94	23.64	8.65	50.1

**Figure 1:**
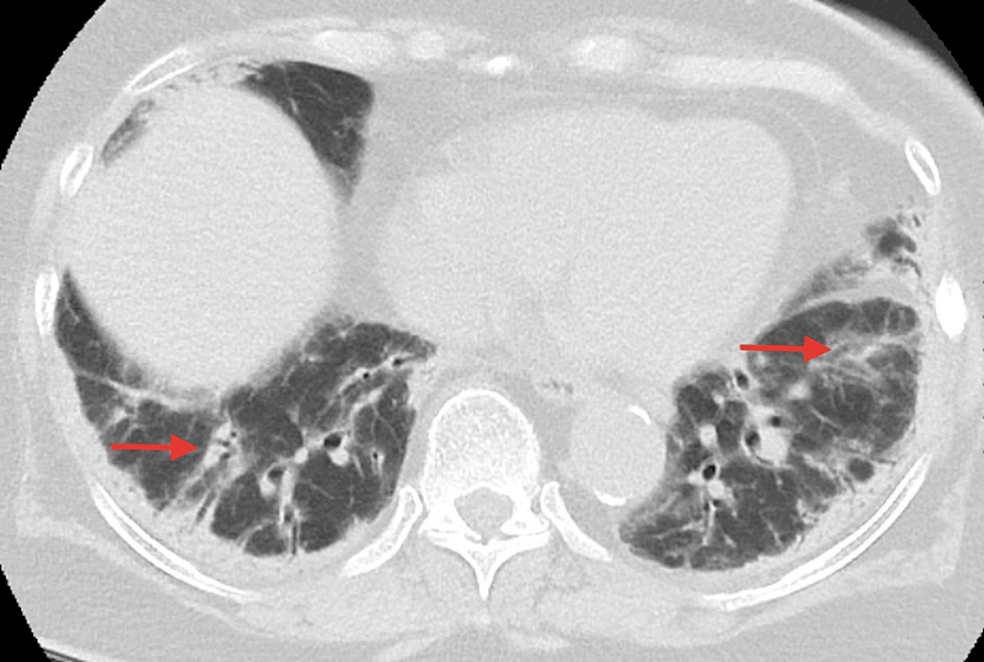
CT chest showing lung bases with bilateral pleural-based ground glass opacities

The CT chest showed pleural thickness with bilateral pleural-based ground glass opacities (Figure 1).

Due to her history of exposure to nitrofurantoin and significant pleural-based disease on imaging, she was started on a prednisone taper for suspected nitrofurantoin-induced ILD. She was prescribed prednisone at 40 mg daily for two weeks, followed by 30 mg daily for two weeks, and then 20 mg daily until the next clinic visit. She was also prescribed pantoprazole, calcium, vitamin D, and Bactrim.

She was seen for a follow-up clinic visit six months after starting the prednisone taper. She reported great improvement in her dyspnea and being able to complete much more of her daily activities. She had repeat PFTs done at that time. Spirometry and lung volumes were normal. Diffusing capacity corrected for hemoglobin was mildly reduced. Compared to the prior study, there was an improvement in FVC (Table 2).

**Table 2:** PFTs at follow-up clinic visit LLN: lower limit of normal; ULN: upper limit of normal; Pre: prior to bronchodilator; Pre%Pred: ratio of patient's actual results compared to predicted normal values; FVC: forced vital capacity; FEV: forced expiratory volume; TLC: total lung capacity; VC: vital capacity; RV: residual volume; DLCO: diffusion capacity of the lungs for carbon monoxide

	LLN	ULN	Pre	Pre%Pred
FVC (L)	1.73	-	2.35	97.1
FEV 0.5 (L)	0.65	-	1.70	113.2
TLC (L)	3.47	5.50	3.71	83.9
VC (L)	1.96	3.09	2.35	92.8
RV (L)	1.09	2.85	1.36	73.7
DLCO mL/(min*mmHg)	12.91	23.61	10.61	61.5

**Figure 2:**
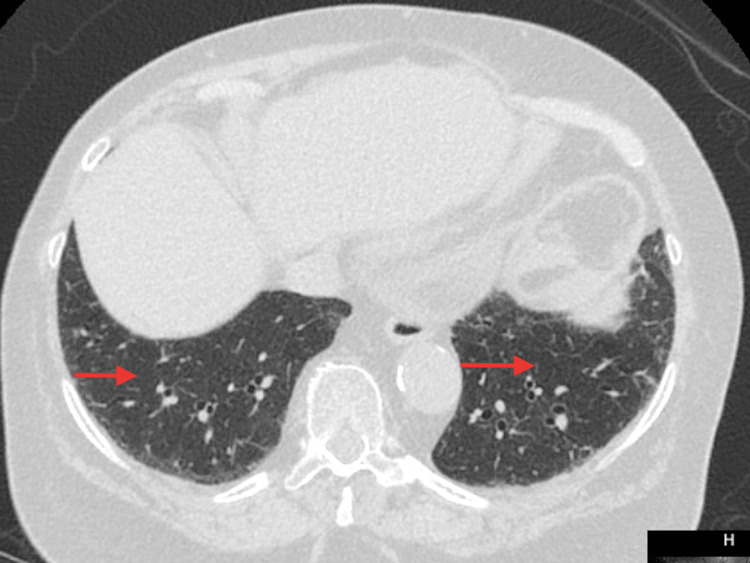
High-resolution CT chest at follow up clinic visit showing near complete resolution of pleural-based lesions and basal ground glass opacities

She also had a high-resolution CT chest, which showed near-complete resolution of pleural-based lesions and basal ground glass opacities (Figure 2).

She was instructed to continue with prednisone 10 mg daily for four weeks, followed by 5 mg daily for two weeks, then 5 mg every other day for two weeks, and finally stop taking prednisone.

## Discussion

Nitrofurantoin first entered the market in 1953 and has been found to have clinical efficacy equivalent to that of trimethoprim-sulfamethoxazole, ciprofloxacin, and amoxicillin for the treatment of UTI [6]. Nitrofurantoin-induced lung injury was first described in 1957 and had an estimated incidence of 1 in 5,000 for acute-severe disease [7]. The chronic form of the disease is predominantly seen in older women who present with dyspnea after being on nitrofurantoin for at least a month, a year, or more [8].

The diagnosis of nitrofurantoin-induced lung injury can be challenging, given the variable clinical presentation. Patients who have acute reactions present with fever, dyspnea, and cough starting from hours to weeks after the first dose. Patients who have chronic reactions present with fatigue, cough, and dyspnea at least one month after the initiation of the antibiotic [7]. The acute phase will show bilateral ground glass opacities on the CT chest, while the chronic phase will show ground glass opacities and fibrosis with no well-defined distribution [8]. Pulmonary function testing should also be obtained. The TLC, or total volume in the lungs at maximal inspiration, as well as the RV, or volume of air remaining in the lungs after maximal exhalation, will both be reduced in the assessment of ILD [9]. Our patient demonstrated evidence of the chronic reaction, with symptoms of fatigue, cough, and dyspnea worsening two months after the initiation of nitrofurantoin, which was prescribed for the prophylaxis of UTIs. She also had radiologic findings consistent with ground-glass opacities and fibrosis. Her PFTs showed a decrease in her TLC and RV. Given her recent diagnosis of COVID-19 five months ago, the diagnosis of nitrofurantoin-induced lung injury may have been missed without a comprehensive history, including a medication review. The histopathological diagnosis in the acute reaction will show mild interstitial inflammation, eosinophilia, focal hemorrhage, and small organizing microthrombi. In contrast, the histopathological diagnosis of the chronic reaction will show interstitial inflammation, fibrosis, thickening of the alveolar septa, and vascular sclerosis [10].

The treatment of acute pulmonary toxicity includes the discontinuation of nitrofurantoin, which results in immediate clinical improvement in the first 24 hours. It has been shown that a short steroid course may be beneficial for patients with severe reactions or those who do not have clinical improvement with discontinuation of the medication [11]. There is no defined evidence showing the benefit of using steroids in addition to discontinuation of the offending agent [12]. We elected to prescribe our patient a prednisone taper in addition to discontinuation of the antibiotic, and she had significant improvement in symptoms, lung volumes, and radiographic findings of restrictive lung disease.

## Conclusions

Cases of nitrofurantoin-induced lung injury have been well documented, but they are still often misdiagnosed, which leads to a delay in treatment. It is critical for clinicians to be aware of this potential adverse effect of an antibiotic that is commonly used for the treatment and prophylaxis of UTIs. There should be suspicion for this diagnosis with any sign of dyspnea occurring after initial or long-term use of nitrofurantoin. The pulmonary toxicity of ILD that could occur is dangerous and potentially fatal if missed. Our patient showed great improvement in her dyspnea as well as marked improvement in restriction on PFTs and near-complete resolution of pleural-based lesions and ground glass opacities on CT chest with discontinuation of nitrofurantoin and initiation of a prednisone taper. This case also shows that there is a need for guidelines to be set for the monitoring of this potential adverse effect.

## References

[1] Guay DR (2001). An update on the role of nitrofurans in the management of urinary tract infections. Drugs.

[2] Lin DC, Bhally H (2007). Nitrofurantoin-induced interstitial lung disease. N Z Med J.

[3] Reynolds TD, Thomas J (2013). Nitrofurantoin related pulmonary disease: a clinical reminder. BMJ Case Rep.

[4] Martin WJ 2nd (1983). Nitrofurantoin. Potential direct and indirect mechanisms of lung injury. Chest.

[5] Bush A (2009). Interstitial lung disease guideline. Thorax.

[6] Huttner A, Verhaegh EM, Harbarth S, Muller AE, Theuretzbacher U, Mouton JW (2015). Nitrofurantoin revisited: a systematic review and meta-analysis of controlled trials. J Antimicrob Chemother.

[7] Tatley M (2002). Pulmonary reactions with nitrofurantoin. Prescriber Update.

[8] Weir M, Daly GJ (2013). Lung toxicity and nitrofurantoin: the tip of the iceberg?. QJM.

[9] Madani Y, Mann B (2012). Nitrofurantoin-induced lung disease and prophylaxis of urinary tract infections. Prim Care Respir J.

[10] Syed H, Bachuwa G, Upadhaya S, Abed F (2016). Nitrofurantoin-induced interstitial pneumonitis: albeit rare, should not be missed. BMJ Case Rep.

[11] Kabbara WK, Kordahi MC (2015). Nitrofurantoin-induced pulmonary toxicity: a case report and review of the literature. J Infect Public Health.

[12] Peall AF, Hodges A (2007). Concomitant pulmonary and hepatic toxicity secondary to nitrofurantoin: a case report. J Med Case Rep.

